# Risk factors for multidrug resistance and mortality in healthcare-associated neonatal gram-negative bloodstream infections

**DOI:** 10.1007/s10096-026-05565-7

**Published:** 2026-06-09

**Authors:** Hatice Turgut, Ramazan Özdemir, Funda Memişoğlu

**Affiliations:** 1 Department of Pediatrics, Division of Neonatology, Inonu University Faculty of Medicine, Malatya, Türkiye; 2 Department of Clinical Microbiology and Infectious Diseases, Inonu University Faculty of Medicine, Malatya, Türkiye

**Keywords:** Neonate, Gram-negative bacterial infections, Multidrug resistance, Neonatal intensive care unit, Appropriate empirical therapy

## Abstract

**Purpose:**

This study aimed to characterize pathogen distribution and antimicrobial resistance patterns of gram-negative bloodstream infections in a tertiary neonatal intensive care unit and to determine which factors were associated with multidrug resistance (MDR) and 28-day mortality.

**Methods:**

In this retrospective cohort study, 197 neonates with healthcare-associated gram-negative bloodstream infections diagnosed between January 2010 and December 2025 were included. Demographic, perinatal, clinical, and microbiological data were extracted from electronic medical records. Factors associated with MDR infection and 28-day mortality were evaluated using univariable and multivariable logistic regression analyses.

**Results:**

Of the 197 neonates, 114 (57.9%) had MDR gram-negative bloodstream infections. *Klebsiella* spp. was the most frequently isolated pathogen (51.3%), followed by *Acinetobacter* spp. (23.9%) and *Escherichia coli* (14.7%). Extended-spectrum beta-lactamase production was detected in 68.6% of isolates, MDR in 57.9%, and carbapenem resistance in 39.1%. *Acinetobacter* spp. showed the highest rates of carbapenem resistance (95.7%) and MDR (97.9%). In multivariable analysis, older postnatal age at infection onset, mechanical ventilation, and previous carbapenem exposure were independently associated with MDR infection, whereas appropriate empirical therapy was protective. Overall, 83 neonates (42.1%) died within 28 days. Mechanical ventilation and inotropic support were independently associated with mortality, while appropriate empirical therapy remained independently protective. MDR status and pathogen distribution were not independently associated with mortality.

**Conclusion:**

Neonatal gram-negative bloodstream infections were characterized by a high burden of MDR. Mortality was more strongly related to indicators of illness severity than to microbiological resistance profiles. Appropriate empirical therapy was protective against both MDR infection and 28-day mortality.

**Supplementary Information:**

The online version contains supplementary material available at https://doi.org/10.1007/s10096-026-05565-7.

## Introduction

Neonatal infections account for more than one-third of neonatal deaths worldwide. Within this spectrum, sepsis is among the leading causes of neonatal mortality and is responsible for more than one million deaths globally each year [1, 2]. In particular, the growing burden of antimicrobial resistance among resistant gram-negative bacteria has become one of the major challenges in neonatal intensive care units, contributing to increased mortality and adverse long-term outcomes [3].

In recent years, the increasing prevalence of multidrug resistance (MDR), particularly among gram-negative bacteria, has further complicated clinical management by reducing the effectiveness of initial therapy. The increasingly frequent reporting of extended-spectrum beta-lactamase (ESBL)-producing Enterobacterales and carbapenem-resistant strains not only limits treatment options but also highlights the importance of center-specific surveillance data [4–7]. A multicenter BARNARDS study clearly demonstrated the high prevalence of resistance to multiple antibiotic classes, as well as cephalosporin and carbapenem resistance determinants, among gram-negative isolates causing neonatal sepsis [5]. Similarly, data from other studies have confirmed a substantial resistance burden among gram-negative neonatal sepsis isolates, particularly against third-generation cephalosporins and aminoglycosides [4]. In NICU cohorts, previous exposure to broad-spectrum antibiotics has been shown to be associated with MDR gram-negative infections, increasing the risk of resistant pathogens and making empirical treatment selection more difficult [8, 9]. Recent clinical data further indicate that high resistance rates to commonly used first-line agents narrow the available treatment options and threaten the effectiveness of initial therap [6, 10].

Another important factor complicating the clinical management of gram-negative bloodstream infections is the marked variability in pathogen distribution across centers and regions. The available literature indicates that *Klebsiella pneumoniae*, *Escherichia coli*, and *Acinetobacter* spp. are the predominant causative organisms in many cohorts, while *Serratia* and *Enterobacter* species have also been reported in some series [4, 5, 11]. The distribution of bacterial pathogens that cause neonatal sepsis also varies over time and across geographic regions, with clear differences in dominant microorganisms between developed and developing countries [12]. This heterogeneous microbiological landscape, together with the high burden of antimicrobial resistance, suggests that a uniform empirical antibiotic approach may not be adequate for every neonatal intensive care unit [4, 5, 13]. Therefore, local surveillance data on pathogen distribution and resistance patterns are essential for guiding empirical treatment policies. Nevertheless, studies that simultaneously evaluate pathogen distribution, resistance profiles, clinical factors associated with multidrug resistance, and determinants of mortality within the same cohort of healthcare-associated gram-negative bloodstream infections remain limited.

Although the literature highlights the overall burden of gram-negative neonatal sepsis, data specific to tertiary-level neonatal intensive care units remain limited. Therefore, the present study aimed to evaluate the distribution of causative microorganisms, antimicrobial resistance characteristics, factors associated with multidrug resistance, and determinants of mortality in healthcare-associated gram-negative bloodstream infections managed in a tertiary-level neonatal intensive care unit.

## Materials and methods

### Study design and setting

This retrospective single-center cohort study was conducted in the neonatal intensive care unit of a tertiary-care university hospital. Neonates who were managed in our unit between January 2010 and December 2025 and who developed healthcare-associated gram-negative bloodstream infection were included in the study. Our center is a tertiary referral neonatal intensive care unit that admits both inborn infants and patients referred from surrounding healthcare facilities.

### Study population

Neonates whose blood culture results were positive for at least one gram-negative bacteria obtained 72 h or more after admission were included in the study. In patients with more than one infectious episode, only the first gram-negative bloodstream infection episode was evaluated to avoid duplicate records. Repeated cultures obtained during the same infectious episode were not considered separate events. Patients with insufficient microbiological data or incomplete clinical records were excluded.

### Data collection

Clinical data were retrospectively obtained from the hospital’s electronic medical records system. Demographic, perinatal, clinical, and microbiological variables were evaluated for each patient.

The demographic and perinatal variables included gestational age, birth weight, very low birth weight (< 1500 g), prematurity (< 37 weeks), small for gestational age (SGA) status, sex, and mode of delivery.

Clinical variables included the need for mechanical ventilation, the presence of a central venous catheter, the administration of total parenteral nutrition, previous exposure to antibiotics and carbapenems, the need for inotropic support, the presence of a surgical condition, the need for dialysis, the postnatal age at the onset of infection (days), the appropriateness of empirical therapy, and mortality occurring within 28 days after infection onset. The results of the microbiological evaluation included the isolated causative microorganisms, resistance phenotypes (extended-spectrum beta-lactamase (ESBL) production, multidrug resistance (MDR), and carbapenem resistance), and antibiotic resistance profiles.

### Definitions

Healthcare-associated gram-negative bloodstream infection was defined as the isolation of a gram-negative bacterium from at least one blood culture obtained 72 h or more after hospital admission. Cases with clinical or microbiological findings suggestive of infection at the time of admission were not considered within this definition.

ESBL production and carbapenem resistance were determined on the basis of routine antibiogram results. Carbapenem resistance was defined as resistance to at least one of the following carbapenems: meropenem, imipenem, or, when applicable, ertapenem.

Multidrug resistance (MDR) was determined for each isolate according to the susceptibility results of the antibiotics tested and was defined as resistance to at least one agent in three or more different antimicrobial classes.For MDR classification, antimicrobial susceptibility results from the routinely tested and organism-appropriate agents available in the microbiology laboratory records were used. The antimicrobial classes considered were aminoglycosides (gentamicin and amikacin), third- and fourth-generation cephalosporins (ceftriaxone, cefepime, and ceftazidime), carbapenems (meropenem, imipenem, and ertapenem), fluoroquinolones (ciprofloxacin), beta-lactam/beta-lactamase inhibitor combinations (piperacillin-tazobactam), polymyxins (colistin), glycylcyclines (tigecycline), and folate pathway inhibitors (trimethoprim-sulfamethoxazole). Classification was based on the susceptibility results of the representative agents available for each isolate in the routine laboratory records; antibiotics not routinely tested or not intrinsically applicable to a given organism were not considered for that isolate.

Mechanical ventilation was defined as the use of invasive respiratory support for at least 72 h before blood culture collection. The presence of a central catheter was defined as the presence of at least one of the following vascular access devices within 72 h before blood culture collection: an umbilical venous catheter, an umbilical arterial catheter, a percutaneous central venous catheter, or any other central vascular access device. Inotropic support was defined as the administration of any inotropic or vasoactive agent within 72 h before blood culture collection. Total parenteral nutrition use was defined as the administration of parenteral nutrition within 72 h before blood culture collection.

Previous antibiotic exposure was defined as the receipt of systemic antibiotic therapy during the same hospital stay before the onset of infection for early-onset neonatal sepsis, clinical sepsis, or gram-positive infections. Previous carbapenem exposure was defined as exposure to meropenem, imipenem, or ertapenem during the same hospitalization before the development of healthcare-associated gram-negative bloodstream infection. Appropriate empirical therapy was defined as the inclusion of at least one antibiotic in the initial empirical treatment regimen to which the causative organism isolated from blood culture was shown to be susceptible in vitro. Mortality was defined as 28-day all-cause mortality, which was defined as death occurring during the same hospitalization within 28 days after the first positive blood culture.

### Microbiological methods

Blood culture samples were obtained under aseptic conditions from neonates with suspected sepsis as part of routine clinical care and were processed in the hospital microbiology laboratory according to standard procedures. The identification of gram-negative bacteria recovered from positive cultures was performed using a MALDI-TOF MS system (bioMérieux, Marcy-l’Etoile, France), and antimicrobial susceptibility testing was carried out using the VITEK 2 automated system (bioMérieux, Marcy-l’Etoile, France). Antibiotic susceptibility results were interpreted according to the clinical breakpoint criteria established by the European Committee on Antimicrobial Susceptibility Testing (EUCAST). ESBL production and carbapenem resistance were determined on the basis of routine antibiogram results; ESBL evaluation was performed only for Enterobacterales isolates. Routine ESBL assessment was not performed for nonfermentative gram-negative bacteria such as *Acinetobacter* spp. and *Pseudomonas* spp. Recurrent isolates belonging to the same infectious episode were not included in the analysis.

### Statistical analysis

Statistical analyses were performed using IBM SPSS Statistics for Windows, version 26.0 (IBM Corp., Armonk, NY, USA). The distribution of continuous variables was assessed using the Kolmogorov–Smirnov and Shapiro–Wilk tests. Continuous variables are presented as medians and interquartile ranges (IQRs), and categorical variables are presented as frequencies and percentages.

Comparisons between groups (MDR vs. non-MDR infections and survivors vs. nonsurvivors) were performed using the Mann–Whitney U test for continuous variables and the chi-square test or Fisher’s exact test for categorical variables, as appropriate.

To identify factors associated with multidrug-resistant (MDR) infection and 28-day mortality, univariable logistic regression analyses were initially performed. Variables with a *p* value < 0.10 in univariable analyses, together with clinically relevant variables, were entered into multivariable logistic regression models. Adjusted odds ratios (aORs) with 95% confidence intervals (CIs) were calculated to identify independent predictors.

Model fit was assessed using the Hosmer–Lemeshow goodness-of-fit test, and the explanatory power of the models was evaluated using Nagelkerke’s R². All the statistical tests were two-tailed, and a *p* value < 0.05 was considered to indicate statistical significance.

### Sample size

As this was a retrospective cohort study, no a priori sample size calculation was performed. All consecutive neonates who met the eligibility criteria and were diagnosed with healthcare-associated gram-negative bloodstream infection during the study period were included in the analysis.

### Ethical considerations

The study protocol was approved by the Institutional Clinical Research Ethics Committee (Approval No: 9782/2026). Owing to the retrospective nature of the study, informed consent was waived. The study was conducted in accordance with the Declaration of Helsinki. All the data were anonymized prior to analysis, and patient confidentiality was strictly maintained.

## Results

Among the 197 neonates included in the study, 114 (57.9%) had multidrug-resistant (MDR) gram-negative bloodstream infections. No significant differences were observed between the MDR and non-MDR groups with respect to gestational age, birth weight, prematurity, SGA status, sex, or postnatal age at infection onset. In contrast, the need for mechanical ventilation was significantly greater in the MDR group (*p* = 0.010), whereas the rate of receiving appropriate empirical therapy was significantly lower (*p* < 0.001). No significant differences were found between the two groups for the other clinical variables. The distribution of microorganisms differed significantly between the groups; *Acinetobacter* spp. was more predominant in the MDR group, whereas *Escherichia coli* and *Serratia* spp. were more frequently identified in the non-MDR group (*p* < 0.001). In contrast, *Klebsiella* spp. were observed at a similar frequency in both groups (Table 1).

**Table 1:** Clinical and microbiological characteristics of neonates with gram-negative sepsis stratified by MDR status

Variable	Total (*n*:197)	MDR (*n* = 114)	Non-MDR (*n* = 83)	*p* value
Gestational age (weeks)^a^	31 (27–36)	31 (27–36)	30 (27–37)	0.783
Birth weight (g)^a^	1440 (980–2550)	1590 (1000–2600)	1400 (975–2500)	0.830
< 1500 g, n (%)	104 (52.8)	58 (50.9)	46 (55.4)	0.528
Prematurity (< 37 weeks), n (%)	152 (77.3)	91 (79.8)	61 (73.5)	0.296
SGA, n (%)	27 (13.7)	15 (13.2)	12 (14.5)	0.793
Male sex, n (%)	108 (54.8)	59 (51.8)	49 (59.0)	0.311
Postnatal age at infection onset, days^a^	11 (7–22)	10 (7–18)	12 (7–24.5)	0.099
Mechanical ventilation, n (%)	102 (51.8)	68 (59.6)	34 (41.0)	0.010
Central catheter, n (%)	103 (52.3)	61 (53.5)	42 (50.6)	0.687
TPN use, n (%)	127 (64.5)	74 (64.9)	53 (63.9)	0.878
Prior antibiotic use, n (%)	177 (89.8)	102 (89.5)	75 (90.4)	0.839
Prior carbapenem use, n (%)	34 (17.3)	21 (18.4)	13 (15.7)	0.613
Inotropic support, n (%)	58 (29.4)	35 (30.7)	23 (27.7)	0.649
Surgical disease, n (%)	55 (27.9)	30 (26.3)	25 (30.1)	0.557
Dialysis requirement, n (%)	19 (9.6)	12 (10.5)	7 (8.4)	0.623
Appropriate empirical therapy, n (%)	120 (60.9)	49 (43.0)	71 (85.5)	< 0.001
Mortality, n (%)	83 (42.1)	51 (44.7)	32 (38.6)	0.386
Microorganism distribution, n (%)				
*Klebsiella spp.*, n (%) *Acinetobacter spp.*, n (%) *Escherichia coli*, n (%) *Pseudomonas spp.*, n (%) *Serratia spp.*, n (%)	101 (51.3) 47 (23.9) 29 (14.7) 10 (5.1) 10 (5.1)	58 (50.9) 46 (40.4) 7 (6.1) 3 (2.6) 0	43 (51.8) 1 (1.2) 22 (26.5) 7 (8.4) 10 (12.1)	< 0.001

Abbreviations: *SGA* small for gestational age, *TPN* total parenteral nutrition, *MDR* multidrug resistance

^a^Data are presented as medians (IQRs). Comparisons were performed using the Mann–Whitney U test for continuous variables and χ² or Fisher’s exact test for categorical variables, as appropriate

MDR was defined as resistance to ≥ 3 antimicrobial classes

Percentages are calculated within each column

Among the 197 gram-negative isolates, the most frequently isolated microorganism was *Klebsiella* spp. (51.3%), followed by *Acinetobacter* spp. (23.9%) and *Escherichia coli* (14.7%). ESBL production was detected in 68.6% of the isolates, multidrug resistance in 57.9%, and carbapenem resistance in 39.1%. When resistance phenotypes were analyzed according to microorganisms, *Acinetobacter* spp. had the highest rates of carbapenem resistance (95.7%) and MDR (97.9%). Although ESBL production was common in *Klebsiella* spp. (78.2%), carbapenem resistance was observed at a relatively low rate (24.8%) (Table 2).

**Table 2:** Distribution of antimicrobial resistance phenotypes among gram-negative isolates in the NICU

Pathogen	*n* (%)	ESBL, *n*/*N* (%)	CR, *n*/*N* (%)	MDR, *n*/*N* (%)
*Klebsiella spp.*	101 (51.3)	79/101 (78.2)	25/101 (24.8)	58/101 (57.4)
*Acinetobacter spp.*	47 (23.9)	NA	45/47 (95.7)	46/47 (97.9)
*Escherichia coli*	29 (14.7)	15/29 (51.7)	2/29 (6.9)	7/29 (24.1)
*Pseudomonas spp.*	10 (5.1)	NA	5/10 (50.0)	3/10 (30.0)
*Serratia spp.*	10 (5.1)	2/10 (20.0)	0/10 (0.0)	0/10 (0.0)
Total	**197 (100.0)**	**96/140 (68.6)**	**77/197 (39.1)**	**114/197 (57.9)**

Abbreviations: *ESBL* extended-spectrum β-lactamase, *CR* carbapenem resistance, *MDR* multidrug resistance, *NA* not applicable

The percentages are presented as n/N (%), where n indicates the number of isolates with the specified resistance phenotype and N indicates the total number of isolates tested within each microorganism group. ESBL testing was not performed for non-Enterobacterales organisms (Acinetobacter spp. and Pseudomonas spp.)

Antibiotic resistance profiles varied markedly across microorganisms. The highest resistance rates were observed among *Acinetobacter* spp. isolates, whose resistance to gentamicin (100%), meropenem (93.6%), ciprofloxacin (97.8%), and piperacillin-tazobactam (97.2%) was particularly high. In *Klebsiella* spp. isolates, resistance was especially pronounced for cephalosporins and trimethoprim-sulfamethoxazole; resistance rates to ceftriaxone and ceftazidime were 79.6% and 80.8%, respectively, whereas meropenem resistance was 15.8%. The overall resistance rates were relatively low in the *Escherichia coli* isolates, while the distribution of resistance was more heterogeneous in the *Pseudomonas* spp. and *Serratia* spp. Among the tested isolates, resistance to colistin and tigecycline was 11.0% and 21.6%, respectively (Table 3).

**Table 3:** Antimicrobial resistance rates among gram-negative pathogens

Antibiotic	Total *n*/*N* (%)	Klebsiella spp. *n*/*N* (%)	Acinetobacter spp. *n*/*N* (%)	E. coli *n*/*N* (%)	Pseudomonas spp. *n*/*N* (%)	Serratia spp. *n*/*N* (%)
Gentamicin	116/173 (67.1)	61/88 (69.3)	47/47 (100.0)	7/26 (26.9)	1/4 (25.0)	0/8 (0.0)
Amikacin	61/196 (31.1)	15/100 (15.0)	44/47 (93.6)	1/29 (3.4)	1/10 (10.0)	0/10 (0.0)
Ceftriaxone	133/178 (74.7)	78/98 (79.6)	32/33 (97.0)	14/29 (48.3)	5/8 (62.5)	4/10 (40.0)
Cefepime	134/179 (74.9)	79/100 (79.0)	30/31 (96.8)	16/29 (55.2)	5/9 (55.6)	4/10 (40.0)
Ceftazidime	136/180 (75.6)	80/99 (80.8)	31/33 (93.9)	17/29 (58.6)	5/9 (55.6)	3/10 (30.0)
Meropenem	62/197 (31.5)	16/101 (15.8)	44/47 (93.6)	1/29 (3.4)	1/10 (10.0)	0/10 (0.0)
Imipenem	65/195 (33.3)	15/100 (15.0)	44/47 (93.6)	1/28 (3.6)	5/10 (50.0)	0/10 (0.0)
Ertapenem	71/181 (39.2)	25/98 (25.5)	44/45 (97.8)	2/28 (7.1)	NA	0/10 (0.0)
Fluoroquinolones						
Ciprofloxacin	87/172 (50.6)	38/86 (44.2)	44/45 (97.8)	4/25 (16.0)	1/8 (12.5)	0/8 (0.0)
Trimethoprim–sulfamethoxazole	100/180 (55.6)	59/97 (60.8)	27/44 (61.4)	14/28 (50.0)	0/1 (0.0)	0/10 (0.0)
Piperacillin–tazobactam	82/176 (46.6)	37/95 (38.9)	35/36 (97.2)	6/26 (23.1)	3/10 (30.0)	1/9 (11.1)
Colistin	9/82 (11.0)	9/31 (29.0)	0/45 (0.0)	0/4 (0.0)	0/2 (0.0)	NA
Tigecycline	16/74 (21.6)	7/29 (24.1)	9/41 (22.0)	0/4 (0.0)	NA	NA

Abbreviations: *NA* not applicable. Data are presented as n/N (%). NA indicates antibiotics not tested or intrinsically inactive against specific organisms (e.g., ertapenem for Pseudomonas spp.). Percentages were calculated within each microorganism group

In the multivariable analysis, later postnatal onset of infection (adjusted OR: 1.04; 95% CI: 1.01–1.07; *p* = 0.021), the need for mechanical ventilation (adjusted OR: 2.46; 95% CI: 1.27–4.79; *p* = 0.008), and prior carbapenem exposure (adjusted OR: 4.11; 95% CI: 1.37–12.29; *p* = 0.012) were independently associated with MDR infection. In contrast, appropriate empirical therapy was identified as an independent protective factor (adjusted OR: 0.10; 95% CI: 0.04–0.21; *p* < 0.001) (Table 4).

**Table 4:** Clinical and microbiological characteristics of neonates with gram-negative bloodstream infection according to MDR status

Variable	Crude OR (95% CI)	*p* value	Adjusted OR (95% CI)	*p* value
Postnatal age at infection (days)	1.01 (0.99–1.03)	0.239	1.04 (1.01–1.07)	0.021
Mechanical ventilation	2.13 (1.20–3.79)	0.010	2.46 (1.27–4.79)	0.008
Prior carbapenem use	1.21 (0.57–2.59)	0.613	4.11 (1.37–12.29)	0.012
Appropriate empirical therapy	0.12 (0.06–0.26)	< 0.001	0.10 (0.04–0.21)	< 0.001

Hosmer–Lemeshow goodness-of-fit test *p* = 0.356; Nagelkerke R² = 0.324. Abbreviations: OR, odds ratio; CI, confidence interval

In the analysis of factors associated with mortality, 28-day mortality occurred in 83 of the 197 neonates (42.1%). The nonsurvivor group had a significantly lower birth weight (*p* = 0.009) and an earlier postnatal age at infection onset (*p* = 0.002). Although gestational age tended to be lower in the nonsurvivor group, this difference did not reach statistical significance (*p* = 0.052). In terms of clinical characteristics, the need for mechanical ventilation (*p* < 0.001), the presence of a central catheter (*p* = 0.006), the use of total parenteral nutrition (*p* = 0.002), the requirement for inotropic support (*p* < 0.001), and prior antibiotic exposure (*p* = 0.034) were significantly more common in the nonsurvivor group. In contrast, surgery was more common among survivors (*p* = 0.047). With respect to microbiological factors, ESBL production (*p* = 0.830), carbapenem resistance (*p* = 0.052), the presence of MDR (*p* = 0.386), and the distribution of causative microorganisms were not significantly associated with mortality (Table 5).

**Table 5:** Clinical and microbiological factors associated with mortality in neonates with gram-negative bloodstream infection

Variable	Survival (*n* = 114)	Nonsurvival (*n* = 83)	*p* value
Gestational age (weeks)^a^	31 (28–37)	30 (26.5–35)	0.052
Birth weight (g)^a^	1575 (1030–2800)	1300 (860–2050)	0.009
< 1500 g, n (%)	55 (48.2)	49 (59.0)	0.134
Prematurity, n (%) (< 37 weeks)	84 (73.7)	68 (81.9)	0.174
SGA, n (%)	13 (11.4)	14 (16.9)	0.271
Male sex, n (%)	63 (55.3)	45 (54.2)	0.884
Postnatal age at infection onset, days^a^	12.5 (9–24)	9 (6–16)	0.002
Mechanical ventilation, n (%)	39 (34.2)	63 (75.9)	< 0.001
Central catheter, n (%)	50 (43.9)	53 (63.9)	0.006
TPN use, n (%)	63 (55.3)	64 (77.1)	0.002
Prior antibiotic use, n (%)	98 (86.0)	79 (95.2)	0.034
Prior carbapenem use, n (%)	21 (18.4)	13 (15.7)	0.613
Appropriate empirical therapy, n (%)	75 (65.8)	45 (54.2)	0.100
Inotropic support, n (%)	10 (8.8)	48 (57.8)	< 0.001
Surgical disease, n (%)	38 (33.3)	17 (20.5)	0.047
Dialysis requirement, n (%)	7 (6.1)	12 (14.5)	0.051
Microbiological factors, n (%)			
ESBL-producing organism, n (%) Carbapenem-resistant organism, n (%) MDR organism, n (%)	85 (74.6) 38 (33.3) 63 (55.3)	63 (75.9) 39 (47.0) 51 (61.4)	0.830 0.052 0.386
*Klebsiella spp*., n (%) *Acinetobacter spp.*, n (%) *Escherichia coli*, n (%) *Pseudomonas spp.*, n (%) *Serratia spp*., n (%)	60 (52.6) 27 (23.7) 13 (11.4) 6 (5.3) 8 (7.0)	41 (49.4) 20 (24.1) 16 (19.3) 4 (4.8) 2 (2.4)	0.654 0.947 0.124 0.889 0.146

*SGA* small for gestational age, *TPN* total parenteral nutrition, *ESBL* extended-spectrum β-lactamase, *MDR* multidrug resistance. MDR was defined as resistance to ≥ 3 antimicrobial classes

^a^Data are presented as medians (IQRs)

In the multivariable analysis, mechanical ventilation was independently associated with mortality (adjusted OR: 3.33; 95% CI: 1.59–6.94; *p* = 0.001). Inotropic support emerged as the strongest independent predictor of mortality (adjusted OR: 11.66; 95% CI: 4.99–27.22; *p* < 0.001). In contrast, appropriate empirical therapy was identified as an independent protective factor against mortality and was associated with an approximately 62% reduction in the odds of death (adjusted OR: 0.38; 95% CI: 0.16–0.86; *p* = 0.020). Prior antibiotic exposure and Acinetobacter infection did not retain independent significance in the multivariable model (Table 6).

**Table 6:** Multivariable logistic regression analysis of factors associated with mortality in neonates with gram-negative bloodstream infection

Variable	Crude OR (95% CI)	*p* value	Adjusted OR (95% CI)	*p* value
Mechanical ventilation	6.06 (3.21–11.42)	< 0.001	3.33 (1.59–6.94)	0.001
Inotropic support	14.26 (6.52–31.17)	< 0.001	11.66 (4.99–27.22)	< 0.001
Prior antibiotic use	3.22 (1.04–10.03)	0.043	2.40(0.60–9.72)	0.217
Appropriate empirical therapy	0.62 (0.34–1.10)	0.101	0.38 (0.16–0.86)	0.020
Acinetobacter infection	1.02 (0.52–1.98)	0.947	0.62 (0.24–1.60)	0.329

Hosmer–Lemeshow goodness-of-fit test *p* = 0.993; Nagelkerke R² = 0.441. Abbreviations: *OR* odds ratio, *CI* confidence interval

Among the 197 neonates, infection-related complications were identified in 100 patients (50.8%). The most common complication was septic shock (18.8%), followed by gastrointestinal complications (11.2%), respiratory complications (9.1%), renal complications (6.6%), and neurological complications (5.1%). Septic shock, respiratory complications, and renal complications were significantly more frequent among nonsurvivors (Supplementary Table S1).

## Discussion

### High burden of MDR, ESBL production, and carbapenem resistance

Neonatal sepsis remains among the leading causes of mortality and serious morbidity in neonatal intensive care units. In this vulnerable patient population, where antibiotic exposure is high and invasive procedures and life-support interventions are frequently used, multidrug-resistant (MDR) gram-negative bacteria (GNB) have become an increasingly important challenge because they complicate empirical treatment selection and make clinical management more difficult. In our study, ESBL production (68.6%), MDR (57.9%), and carbapenem resistance (39.1%) were strikingly high among gram-negative bloodstream infections.

In a study by Ballot et al., which evaluated MDR *Enterobacteriaceae* infections, the ESBL production rate was reported to be 71%, and this mechanism was identified as one of the major drivers of resistance development [14]. Similarly, in our cohort, ESBL production, which is particularly prominent in *Klebsiella* spp., appears to be one of the main mechanisms shaping the resistance pattern. These findings may lead to selective pressure that favors the use of carbapenems in empirical therapy; consequently, increased use of broad-spectrum antibiotics may further increase resistance selection [2, 8, 14, 15].

The marked variation in MDR rates reported across centers is also noteworthy. A study from Jordan reported an MDR rate of 69%, whereas two studies from Ethiopia reported rates of 55% and 88%, respectively. In contrast, Tsai et al. reported an MDR gram-negative bacteria rate of 18.6% in a NICU cohort from Taiwan [8, 16–18]. These differences may be related to local variability in pathogen distribution, resistance patterns, antibiotic use practices, and infection control measures. Therefore, regular reassessment of empirical antibiotic policies and infection control strategies based on local surveillance data is critically important in neonatal intensive care units.

### Pathogen distribution and resistance profile

In our study, *Klebsiella* spp. was the most frequently isolated pathogen, followed by *Acinetobacter* spp. and *Escherichia coli*. When evaluated by pathogens, the MDR rates were 97.9% for *Acinetobacter* spp., 57.4% for *Klebsiella* spp., and 24.1% for *E. coli*. These findings indicate that the burden of resistance in neonatal gram-negative bloodstream infections is not distributed uniformly across pathogens but instead shows a distinct pathogen-specific pattern. In a series reported from Jordan, the MDR rates for the same pathogens were 82%, 54%, and 38%, respectively, whereas a review from South Asia reported rates of 78.7%, 70.7%, and 54%, respectively, and another cohort reported rates of 47%, 66%, and 32%, respectively [10, 16, 18]. These comparisons support the view that *Acinetobacter* spp. and *Klebsiella* spp. are among the principal contributors to the resistance burden across different centers. The high MDR rate observed in *Klebsiella* spp., when considered together with its high isolation frequency, suggests that this organism represents one of the main drivers of resistance complicating clinical management in our unit. In contrast, the relatively low MDR rate in *E. coli* indicates that the resistance burden is not equally distributed among all gram-negative pathogens. Nevertheless, these findings also show that *E. coli* still has clinically relevant resistance potential that should not be overlooked.

### Risk factors associated with MDR infection

In our study, no significant differences were observed between the MDR and non-MDR groups with respect to basic perinatal characteristics. In contrast, the need for mechanical ventilation was greater in the MDR group, and multivariable analysis revealed that both mechanical ventilation and prior carbapenem exposure were independently associated with MDR infection. These findings suggest that mechanical ventilation may not merely reflect disease severity but may also contribute to the risk of MDR gram-negative infection through more intensive invasive care practices, greater exposure to the intensive care environment, and increased contact with resistant hospital flora. Similarly, prior carbapenem use appears to be an important risk factor for MDR infection through the suppression of susceptible strains and the promotion of the selection of resistant gram-negative pathogens. Consistent with these findings, previous studies have reported that exposure to third-generation cephalosporins and carbapenems is among the independent risk factors for MDR gram-negative bloodstream infections [8, 14, 19].

Findings in the literature regarding the association between mechanical ventilation and MDR are heterogeneous, while some studies have identified it as an independent risk factor, others have not demonstrated a significant association in their cohorts [8, 20–22]. This variation across studies suggests that the relationship between mechanical ventilation and MDR may be influenced by factors such as patient population characteristics, disease severity, intensity of care, and the multivariable modeling approach used. Overall, these findings indicate that MDR infection is closely related not only to the microbiological characteristics of the pathogen but also to patient- and care-related factors, including the intensity of clinical care and prior antimicrobial exposure.

### Factors associated with mortality

In our study, the nonsurvivor group had lower birth weight, earlier postnatal onset of infection, and higher rates of mechanical ventilation, central catheter use, total parenteral nutrition, and especially inotropic support. These findings suggest that mortality is primarily associated with variables reflecting clinical severity and the need for intensive care support. Multivariate analysis further revealed that mortality was concentrated particularly among patients with respiratory and hemodynamic instability. The independent association between inotropic support and mortality suggests that hemodynamic compromise is among the strongest indicators of poor prognosis in this patient population. In contrast, the lack of an independent association between *Acinetobacter* infection and mortality, as well as the loss of significance of prior antibiotic exposure in the multivariable model, suggests that these variables are not prognostic determinants on their own and that their effects may largely be explained by other factors reflecting overall clinical severity.

Our findings are broadly consistent with those of Thatrimontrichai et al., who evaluated 30-day mortality in patients with gram-negative bloodstream infections. In that study, nonsurvivors were more likely to have lower gestational age and birth weight, earlier onset of sepsis, higher rates of mechanical ventilation and central catheter use, and more frequent MDR gram-negative infection and inadequate empirical therapy; septic shock and failure to achieve microbiological clearance emerged as the main independent risk factors [9]. In another study evaluating mortality within 72 h, younger gestational age, the development of acute kidney injury, and the need for inotropic support were identified as independent risk factors [21].

In our cohort, pathogen distribution and resistance patterns did not differ significantly with respect to mortality. ESBL positivity, the MDR phenotype, and the pathogen distribution were similar between survivors and nonsurvivors, whereas only carbapenem resistance tended to be higher in the nonsurvivor group. These findings suggest that although resistant microorganisms remain clinically important, the risk of mortality cannot be explained solely by the microbiological resistance profile. Consistent with this, Ballot et al. reported no association between ESBL production or carbapenem resistance and mortality [14]. In contrast, Thatrimontrichai et al. reported a higher rate of MDR-GNB among nonsurvivors (75.7% vs. 61.7%, *p* = 0.047), although this association did not retain independent significance in the multivariable analysis [9]. Similarly, although several studies have reported that the MDR-GNB phenotype is more frequent in the nonsurvivor group, this increase has not been shown to be an independent predictor of mortality in multivariable models [8, 23].

The literature also contains findings suggesting that certain pathogens may have a more pronounced effect on mortality. In one study evaluating late-onset sepsis, *Pseudomonas aeruginosa* infection was identified as an independent risk factor for in-hospital mortality [23]. In another study, the highest mortality rate was observed among neonates with *Serratia marcescens* bloodstream infection (55.2%, *p* < 0.017), suggesting that the microbiological pathogen itself may influence the clinical course [14]. In addition, another study revealed that *P. aeruginosa* and ESBL-producing bacteria were more likely to be associated with severe disease or early death [24]. However, the fact that this relationship has not been observed to the same extent in every cohort suggests that mortality should be evaluated not only in terms of the causative microorganism and resistance phenotype but also together with disease severity and accompanying clinical factors.

### Role of appropriate empirical therapy

Our study demonstrated that appropriate empirical antibiotic therapy is among the key determinants of clinical outcomes in neonatal gram-negative bloodstream infections. The lower rate of appropriate empirical therapy observed in the MDR infection group suggests that the initial treatment in these patients is more likely to inadequately cover the causative pathogen. In contrast, the independent protective effect of appropriate empirical therapy on mortality highlights the critical importance of initiating effective treatment early in the course of infection. Therefore, although the MDR phenotype was not directly associated with mortality, it may still adversely affect the clinical course indirectly by reducing the likelihood of receiving effective treatment at the outset.

These findings are also consistent with the literature. In a study evaluating neonates with MDR gram-negative bloodstream infections, inadequate empirical antibiotic therapy was reported to be more frequent among nonsurvivors, whereas microbiological cure was significantly more common among survivors [19]. Similarly, another study revealed that inadequate initial empirical therapy for gram-negative bloodstream infections was associated with increased rates of major organ damage, infectious complications, and overall mortality [24]. Studies examining nosocomial bloodstream infections in neonatal intensive care units have likewise reported an association between inadequate empirical therapy and increased mortality [25, 26].

Taken together, these findings suggest that the MDR phenotype may contribute to adverse clinical outcomes less through a direct effect on mortality and more by reducing the likelihood of timely administration of appropriate therapy.

Our study has several limitations. Its retrospective, single-center design may limit the generalizability of the findings. In addition, changes in infection control practices, antimicrobial use policies, and supportive care strategies over the long study period may have influenced the results. Another limitation is the lack of molecular confirmation of resistance mechanisms. Furthermore, mortality was assessed as 28-day all-cause mortality rather than infection-attributable mortality. In this vulnerable neonatal population, mortality may have resulted not only from the direct effects of bloodstream infection itself, but also from infection-related clinical deterioration through the exacerbation of underlying comorbidities and coexisting clinical conditions. Nevertheless, the study has several major strengths, including the presentation of long-term data on gram-negative bloodstream infections from a tertiary neonatal intensive care unit, the combined evaluation of microbiological and clinical outcomes, and the assessment of independent associations of MDR infection and mortality through multivariable analyses. The fact that appropriate empirical antibiotic therapy was examined in relation to both MDR infection and mortality further strengthens the clinical relevance of the findings.

**In conclusion**, this study revealed that gram-negative bloodstream infections in a tertiary-level neonatal intensive care unit were characterized by high burdens of MDR, ESBL production, and carbapenem resistance, with marked variation in resistance burden according to the causative pathogen. Moreover, mortality appeared to be related not only to the microbiological resistance profile but also primarily to clinical severity, the need for organ support, and the ability to initiate appropriate empirical therapy at the outset. The independent protective effect of appropriate empirical antibiotic therapy underscores the critical importance of early and effective treatment selection for prognosis. Therefore, in the management of neonatal gram-negative bloodstream infections, empirical treatment strategies based on local resistance patterns, rational antimicrobial stewardship policies, and effective infection control measures are essential not only for reducing the burden of resistance but also for improving survival.

## Supplementary Information

Below is the link to the electronic supplementary material.

Supplementary Material 1 (DOCX 14.9 KB)

## Data Availability

The datasets generated during and/or analysed during the current study are not publicly available due to patient confidentiality and institutional restrictions, but are available from the corresponding author on reasonable request.
