# Supplementary material for: Risk factors for multidrug resistance and mortality in healthcare-associated neonatal gram-negative bloodstream infections
Source: Eur J Clin Microbiol Infect Dis. 2026 Jun 9;45(9):2839–49. doi: 10.1007/s10096-026-05565-7 (PMC13593627; doi:10.1007/s10096-026-05565-7)
Supplement: Supplementary file 1 [file 10096_2026_5565_MOESM1_ESM.docx]

**Supplementary Table S1.** Comparison of infection-related complications between survivors and nonsurvivors among neonates with gram-negative bloodstream infection

| **Variable** | **Total (n=197)** | **Survivors**  **(n=114)** | **Nonsurvivors**  **(n=83)** | **p** |
| --- | --- | --- | --- | --- |
| **Any complication, n (%)** | 100 (50.8) | 35 (30.7) | 65 (78.3) | <0.001 |
| **Septic shock, n (%)** | 37 (18.8) | 3 (2.6) | 34 (41.0) | <0.001 |
| **Gastrointestinal, n (%)** | 22 (11.2) | 15 (13.2) | 7 (8.4) | 0.299 |
| **Respiratory complications, n (%)** | 18 (9.1) | 6 (5.3) | 12 (14.5) | 0.027 |
| **Neurological complications, n (%)** | 10 (5.1) | 8 (7.0) | 2 (2.4) | 0.196 |
| **Renal complications, n (%)** | 13 (6.6) | 3 (2.6) | 10 (12.0) | 0.009 |
